# The Global Fight against the Stigma of Schizophrenia

**DOI:** 10.1371/journal.pmed.0020136

**Published:** 2005-07-26

**Authors:** Nadia Kadri, Norman Sartorius

## Abstract

Stigma attached to mental illness is the greatest obstacle to the improvement of the life of people with mental illness and their families. A global campaign hopes to remove this obstacle.

## Abstract 2

We must dispel the myths and misunderstandings surrounding this illness

The stigma attached to mental illness is the greatest obstacle to the improvement of the lives of people with mental illness and their families [1]. Such stigma results in (1) a lower priority for mental health services, (2) difficulty getting staff of good quality to work in these services, (3) continuing problems in finding employment and housing for people who have had an episode of mental disorder, (4) the social isolation of people who suffer from mental illness and their families, and (5) poorer quality of care for physical illnesses occurring in people diagnosed as having had psychiatric illnesses [1]. These effects of stigma are true for all mental disorders, and in particular, for schizophrenia.

The history of the stigmatisation of mental illness is long, but it is probable that intolerance to mental abnormality (and the rejection of people who had it) has become stronger in the past two centuries because of urbanisation and the growing demands for skills and qualifications in almost all sectors of employment. This, however, is only part of the story: mental illness is also linked to stigmatisation, discrimination, and intolerance in rural settings and in all countries, regardless of their level of industrialisation and sophistication of labour. Recent studies carried out in developing countries confirm that this stigma is universal [1]—indeed it is fair to say that stigma is attached to mental illness in different socio-cultural settings throughout the world, and that it is growing in strength and in its negative consequences.

## Programmes against Stigma

A number of programmes to diminish the stigma related to mental illness and its consequences have been started in recent years. Among those well known are campaigns undertaken in Australia, the United Kingdom, and Sweden—for example, “Changing Minds,” an anti-stigma campaign, was launched in 1998 by the United Kingdom Royal College of Psychiatrists (www.rcpsych.ac.uk/campaigns/cminds/index.htm). A major international effort is The Global Programme against Stigma and Discrimination Because of Schizophrenia, launched by the World Psychiatric Association (WPA) in 1996 [2].

**Figure 1:**
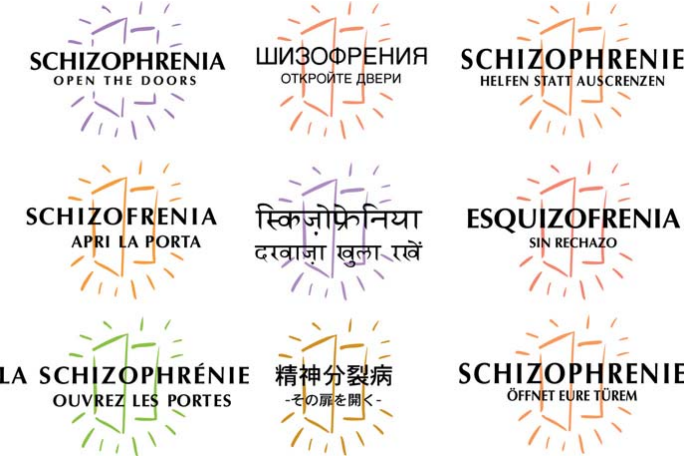
The Logo of Open the Doors, in Nine Different Languages

The WPA programme, known as “Open the Doors” (see www.openthedoors.com and Figure 1), has five important characteristics that distinguish it from other previously developed programmes. First, it is an international and collaborative programme. Second, it is conceived as a long-term programme rather than as a campaign. Third, it involves family and patient organisations as well as governments, community agents, and health services at all stages of the programme, from its planning to its evaluation. Fourth, it emphasises the need for sharing experience and information obtained in the course of the programmes among all concerned, within and between countries. Finally, and perhaps most importantly, the programme's targets are selected on the basis of a process of consultation with people who have schizophrenia and their families rather than on the basis of theoretical constructs. This means that the targets of the programmes in different countries (and even in different regions of the same country) vary. It also means that the forces uniting the programme are shared convictions about the principal and overall goals of the programme rather than an imposed and artificial uniformity of specific short-term objectives.

The WPA programme has already involved some 18 countries as follows: Austria, Brazil, Canada, Chile, Egypt, Germany, Greece, India, Italy, Japan, Morocco, Poland, Romania, Slovakia, Spain, Turkey, the UK, and the United States. It is likely that other countries, for example Zambia and the Czech Republic as well as others, will join it in the years to come.

## What Has the WPA Programme Achieved?

To reach the main goal of the programme—the reduction or elimination of stigma and its consequences—the participating sites have undertaken studies aimed at a better understanding of the causes of stigma, its mechanism of action, and the factors that increase or lessen it [3,4]. Participating sites have also implemented an array of measures (Box 1) that were selected taking into account the country and its specificities.

**Box 1. Measures Implemented by Participating Sites**

- provision of information about schizophrenia, through the programme's websites, meetings, books, articles in health journals, newspapers, lectures, and congresses
- introduction of specific legislation or rules for the behaviour of selected target groups (e.g., health staff in emergency services)
- organisation of artistic and cultural activities
- support for the development of (physical) health services for people with mental disorders
- introduction of anti-stigma activities into the training of different types of professionals (e.g., psychiatrists, police officers, teachers)

## Principles Guiding the Interventions

### Work with patients and relatives

A central priority must be to boost patients' (and families') self-esteem and self-respect. This facilitates patients' socialisation, their active participation in the treatment and rehabilitation process, and their motivation for better personal care.

### Work with members of the health professions

Emphasis has been placed on the fact that health workers can do a great deal (as individuals) to prevent or diminish stigmatisation by: (1) helping their patients maintain self-esteem, (2) developing and implementing the plan of treatment together, (3) being constantly aware of the danger of labelling, which might harm their patients, (4) ensuring that they have respected their patients' priorities rather then placing these priorities below those of the health care system, (5) working with families (learning from their experience and providing them with practical and useful information), and (6) in society, acting as advocates and models of tolerance and acceptance of people with mental illness [5].

### Work with health authorities

Emphasis has been placed on the need to re-examine and improve legislation and procedures governing the health system to avoid its stigmatising potential.

### Work with journalists and other media professionals

Journalists have been engaged in fighting stigma through better reporting about mental illness and about people with mental illness. In Ireland and the Philippines, for example, journalists have been led by the anti-stigma programme to the formulation of a voluntary code of non-stigmatising reporting.

### Work with the general public

The focus here has been on a change in behaviour rather than only a change of attitudes.

The sites participating in the programme are learning from each other through contacts, visits, and the exchange of information. In addition, the programme has identified certain strategic directives that have been incorporated into the rules for new programmes. On the basis of experience, the key documents of the programme—a step-by-step guide on programme development and the manual attached to it—are constantly updated and improved. Many of these documents are available on the programme's website (www.openthedoors.com).

## Measuring Success, Overcoming Obstacles

The success of the WPA programme is evaluated at country level and with direct reference to the targets identified by patients and their families as being particularly important for them. This is being done by focus group explorations of the experiences patients and families have had since the programme in their country began. Surveys of attitudes before the programme and during its course have been done in some countries (e.g., Canada) [6–9]. In addition, there are general indications of the success of the programme, including the increasingly wide use of programme materials, continuing collaboration among the sites, the number of publications and requests for presentation of the programme by both professional and nonprofessional organisations. A detailed description of these matters and references to publications from the participating sites are being published [3].

The main obstacle to success is the fact that changes in attitudes and behaviour take time. Continuous repetition of action and financial support have to be maintained over years—despite the fact that, in the beginning, anti-stigma programmes often produce only meagre results. Maintaining the motivation of all concerned over many years is very difficult. The programme also needs the lasting involvement of all structures of the health system (and of other social services), which must see the fight against stigma as one of their permanent and essential tasks.

## Conclusion

In the descriptions of work done in the sites participating in the WPA Global Programme against Stigma and Discrimination Because of Schizophrenia, there are many examples of actions that have contributed to the lessening of stigma or to the prevention of its consequences. These examples underline the three basic principles that should be kept in mind when fighting stigma.

First, the fight against stigma is a priority because stigmatisation is a major obstacle to any progress in the field of mental health. Second, programmes against stigma and discrimination should select their targets and evaluate their success with the active and concrete involvement of people with mental illness and their families. Finally, each of us, whether part of a major programme or alone, can do something to diminish or avoid stigmatisation by mental illness. It is just as important to ask what we can do ourselves to diminish stigmatisation as it is to urge others to do something about it.

## Abbreviation

WPA: World Psychiatric Association
